# Optilume® for Urethral Strictures: A Comprehensive Review

**DOI:** 10.7759/cureus.82984

**Published:** 2025-04-25

**Authors:** Diogo Carmali, Sónia Ramos, Sara Duarte, Eduardo Felício, Guilherme Bernardo, Filipe Gaboleiro, André Pita, Fernando Ferrito

**Affiliations:** 1 Urology, Unidade Local de Saúde de Amadora/Sintra (ULS Amadora/Sintra), Lisbon, PRT

**Keywords:** drug-coated balloon, optilume, paclitaxel-coated balloon, urethral stricture, urological devices

## Abstract

Urethral stricture disease treatment presents significant challenges due to high recurrence rates and limited minimally invasive options. This review evaluates the efficacy, safety, and clinical applicability of the Optilume® drug-coated balloon, which combines mechanical urethral dilation with paclitaxel delivery, as an innovative alternative to traditional treatments.

A narrative review was conducted using PubMed, focusing on studies published in the last decade addressing the use of Optilume® for urethral strictures. Keywords included "urethral stricture", "Optilume", and "drug-coated balloon". Data from clinical trials (ROBUST I and III) and real-world studies were synthesized to assess functional and anatomical success, patient outcomes, and safety profiles.

Optilume® demonstrated high efficacy in recurrent anterior urethral strictures. ROBUST I showed a 58% functional success rate at five years, with significant improvements in Qmax (5 to 19.9 mL/s) and International Prostate Symptom Score (IPSS) (25.2 to 7.2). The randomized ROBUST III trial reported superior freedom from reintervention compared to standard treatments (77.8% vs. 23.6% at two years). Real-world studies confirmed these findings across diverse patient populations, including those with complex strictures. Immediate- and short-term postoperative complications were mild and infrequent, with no significant impact on erectile function.

Optilume® offers a safe, effective, and minimally invasive solution for urethral stricture management, bridging the gap between endoscopic treatments and urethroplasty. Its advantages include reduced recurrence, shorter recovery times, and preserved sexual function. Future studies should explore its role in treatment-naïve and complex cases to expand its clinical applicability.

## Introduction and background

Urethral stricture disease presents a significant clinical challenge due to its high recurrence rates and the limited availability of effective, minimally invasive treatment options. Affecting approximately 0.6% of men during their lifetime, this condition often requires repeated interventions such as dilations or endoscopic procedures, which frequently lead to restenosis and a need for multiple treatments over time [1]. While urethroplasty remains the gold-standard surgical option, its adoption is limited by its complexity and need for specialized technical expertise, prolonged recovery times, and potential complications [2]. These challenges have prompted the search for alternative therapies that can provide durable outcomes with a lower procedural burden.

The Optilume® drug-coated balloon (DCB) introduces an innovative approach to urethral stricture management by combining mechanical dilation with the localized delivery of paclitaxel, an antiproliferative agent that targets the primary mechanisms of stricture recurrence. 

This review evaluates the current evidence on the efficacy, safety, and clinical applicability of Optilume® as an alternative to conventional treatments. By synthesizing data from clinical trials and real-world studies, it aims to provide clinicians with an updated perspective on this emerging technology and its potential to bridge the gap between endoscopic management and urethroplasty.

## Review

Materials and methods

A narrative review was conducted to assess the efficacy and safety of Optilume® in treating urethral strictures. A comprehensive search of PubMed was performed in December 2024 using the Medical Subject Heading (MeSH) terms "urethral stricture" and "Optilume". Additional free-text searches were carried out using terms such as "bulbar urethral stricture", "drug-coated balloon", "endourethral balloon dilation", and "urethral stents". Only studies published in English and focusing on the use of Optilume® for urethral stricture treatment were included. Studies from the past 10 years were prioritized, but earlier studies were also considered if deemed relevant. A total of four studies were identified and reviewed in this study, including one randomized controlled trial. 

Mechanism of action of paclitaxel

The Optilume® DCB represents a novel approach for treating urethral strictures by combining mechanical dilation with localized drug delivery. Paclitaxel, an antiproliferative agent extensively used in oncology and endovascular procedures, plays a central role in this approach. It prevents fibroblast proliferation by stabilizing microtubules during mitosis, thereby addressing the primary drivers of scar formation and stricture recurrence. Additionally, paclitaxel exhibits anti-inflammatory properties, further reducing post-procedural inflammation and minimizing the risk of restenosis. The localized delivery of paclitaxel ensures high drug concentrations at the stricture site with minimal systemic exposure, optimizing both efficacy and safety [3].

Procedure description

The Optilume® procedure involves a series of well-defined steps to ensure the safe and effective treatment of urethral strictures. The process begins with patient preparation, which includes positioning the patient in lithotomy and administering either intraurethral lidocaine or spinal anesthesia, following standard protocols for endourological procedures. Next, the appropriate balloon catheter must be selected, typically one with a 30 Ch diameter, slightly larger than the adjacent healthy urethra. The length should exceed the total stricture length by at least 1 cm. While fluoroscopy is not essential during the procedure, it may be used if preoperative imaging (cystoscopy, retrograde urethrography, or voiding cystourethrography) has not provided sufficient detail.

The procedure continues with the insertion of a guidewire. A cystoscope, rigid or flexible, is introduced, and a 0.038" guidewire is passed through its working channel, ensuring the flexible tip reaches the bladder. If the stricture is too narrow to allow passage, tools such as filiforms, followers, or Pollack ureteral catheters can be used. Once the guidewire is in place, the Optilume® DCB is introduced under direct vision through the cystoscope or alongside it (if the cystoscope is not compatible with the balloon) and carefully advanced to span the stricture. The balloon should extend approximately 0.5 cm beyond the distal edge of the stricture, with its midpoint centered on the narrowed segment, ensuring stability and effective dilation. If fluoroscopy is employed, the balloon's radiopaque markers should be checked to confirm correct alignment, clearly delineating the stricture's boundaries.

The balloon is then inflated gradually to the recommended burst pressure using a sterile solution of saline or contrast. Inflation with air, carbon dioxide, or other gases is strictly contraindicated. Gentle tension is applied to prevent migration, and if any displacement occurs, inflation should be paused, the balloon repositioned, and the procedure resumed only after verifying proper alignment. Once in place, dilation and drug delivery are carried out by maintaining inflation for at least five minutes. In the case of particularly dense or fibrotic strictures, a longer inflation period may be warranted, based on surgical judgment.

Upon completion of drug delivery, the balloon is deflated by aspiration. If resistance is encountered during removal, gentle rotation of the catheter can help collapse the balloon around the shaft, facilitating smooth extraction. Finally, a urethral catheter (typically 12-16 Ch) is inserted to maintain urethral patency and support tissue healing [4].

**Figure 1:**
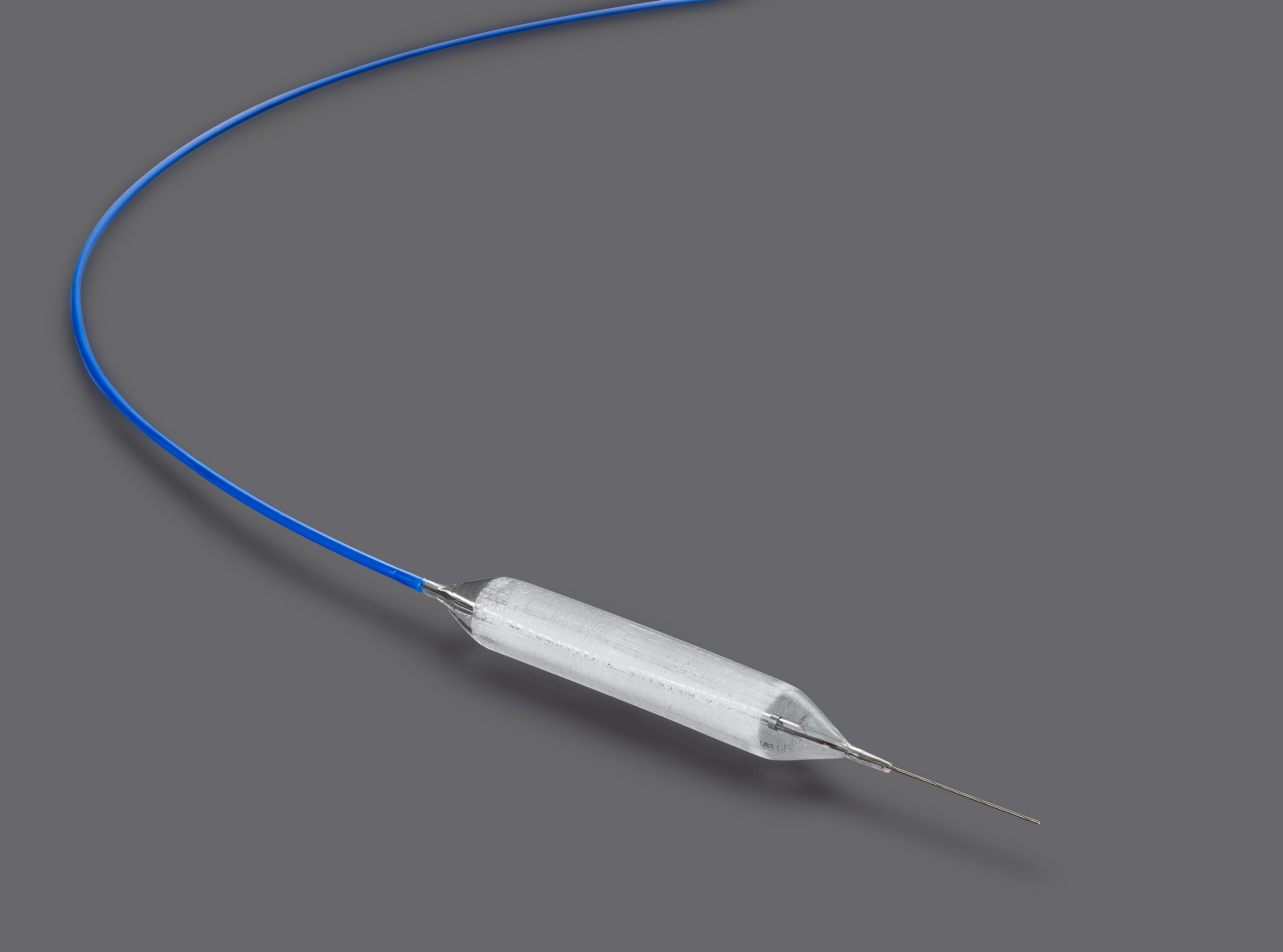
Optilume® product image Image courtesy of Laborie, published with permission

**Figure 2:**
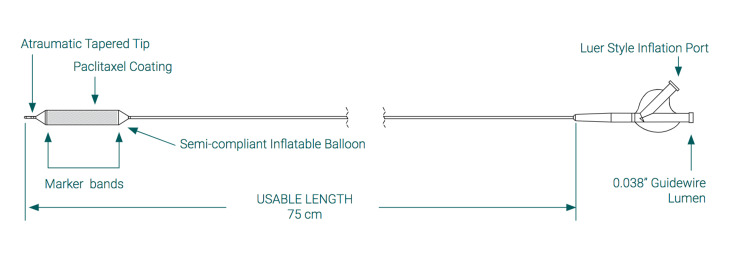
Optilume® schematic drawing Image courtesy of Laborie, published with permission

Below, Figure 1 shows the Optilume® product image, and Figure 2 illustrates a schematic drawing of the device. Both were kindly provided by Laborie (Portsmouth, New Hampshire). 

Tips for the optimal use of the Optilume® device

Several technical considerations can help optimize outcomes when using a DCB for urethral strictures. First, it is essential to dilute the contrast medium with sterile water or saline by at least 50% when filling the inflation device. The use of undiluted contrast increases the viscosity of the solution, which can hinder balloon deflation and compromise the procedure.

Pre-dilation or direct visual internal urethrotomy (DVIU) prior to balloon inflation may be particularly useful in cases of dense or fibrotic strictures, as it facilitates balloon passage and improves treatment efficacy. This preparatory step can enhance the uniformity of balloon expansion and promote more effective drug delivery.

To ensure optimal drug delivery, the DCB should remain in the urethra and hydrate for a minimum of 60 seconds before inflation. During the inflation phase, it is important to monitor the inflation pressure closely. After reaching the recommended final pressure, the pressure gauge should be observed regularly, as stricture tissues often relax during the procedure, leading to pressure drops. In such cases, reinflation may be required to maintain the rated burst pressure throughout the dilation period.

Finally, to minimize the risk of drug washout or mechanical disruption, it is advised to avoid moving the cystoscope through the treated area or activating irrigation flow once the dilation is complete. This helps preserve the drug coating on the urothelium and supports the therapeutic effect of the DCB [4].

ROBUST I: Long-term outcomes of Optilume® for recurrent bulbar strictures

The ROBUST I study was a single-arm, open-label, multicenter, prospective trial designed to evaluate the safety and efficacy of the Optilume® DCB in treating recurrent bulbar urethral strictures. Conducted across four centers in Latin America, the study enrolled 53 men who had experienced at least one prior failed treatment, such as DVIU or urethral dilation. The primary objective was to determine whether Optilume® could achieve sustained stricture-free rates while improving urinary function and quality of life [5].

Selected patients were adult men with a single bulbar urethral stricture of <12 Ch in diameter and ≤2 cm in length (confirmed by retrograde urethrography or cystoscopy), a history of 1-4 prior endoscopic treatments, an International Prostate Symptom Score (IPSS) of ≥13, and a peak urinary flow (Qmax) of <10 mL/s. Exclusion criteria included the following: history of stone passage within six months, lichen sclerosus, chronic kidney disease (creatinine >2 mg/dL), pelvic radiation, penile prosthesis, artificial urinary sphincter, prior urethroplasty, radical prostatectomy, or recent intradetrusor onabotulinumtoxinA injections.

Before Optilume® treatment, strictures were predilated with either DVIU or an uncoated balloon dilator, as determined by the surgeon. Post-procedural follow-up occurred at days 5 and 14, followed by evaluation at months 1, 3, and 6 and then annually for up to five years.

The study's primary endpoints focused on both functional and anatomic success. Functional success was defined as a ≥50% improvement in IPSS compared to baseline without the need for retreatment, while anatomic success was measured by maintained urethral patency confirmed through imaging or cystoscopy during follow-up. Secondary endpoints included improvement in Qmax, reduction in IPSS scores, and patient-reported quality of life measures (e.g., International Index of Erectile Function (IIEF) and Urethral Stricture Surgery Patient-Reported Outcome). The primary safety endpoint was defined as the absence of treatment-related serious adverse events, such as fistula formation, persistent de novo urinary incontinence, or urethral burst.

Out of the 53 enrolled participants, 29 completed the five-year follow-up without the need for retreatment. The remaining patients exited the study due to either a need for retreatment, consent withdrawal, adverse events, or loss to follow-up.

At the five-year follow-up, functional success was achieved by 58% of patients, who required no further interventions. Anatomic success, assessed only at the six- and 12-month follow-ups, was observed in 70% of patients. Freedom from reintervention was estimated at 71.7% based on the Kaplan-Meier analysis, with most failures occurring within the first year.

The mean Qmax significantly increased from 5 mL/s at baseline to 17 mL/s at one year, further stabilizing at 19.9 mL/s by year 5 (p<0.01). Similarly, IPSS scores decreased from a pre-procedural mean of 25.2 to 7.2 at five years (p<0.001), indicating sustained symptomatic relief. Post-void residual volume (PVR) also improved, dropping from 141.4 mL to 59.5 mL (p<0.01). In terms of patient-reported outcomes, the mean Urethral Stricture Surgery Patient-Reported Outcome Measure scores improved from 15.9 to 3.3 over five years.

Regarding safety, a total of 93 mild to moderate adverse events were reported, with only 15 attributed to the device or procedure. Importantly, no serious treatment-related adverse events were documented. IIEF scores remained stable throughout the follow-up period, indicating no impact on erectile function [5].

Mahenthiran et al.: Real-world success with Optilume®

The retrospective study by Mahenthiran et al. evaluated the short-term efficacy and safety of the Optilume® DCB in the treatment of urethral strictures measuring less than 2 cm, regardless of their location or prior interventions. Conducted from November 2022 to August 2023, the study followed patients for up to nine months post-procedure, through medical consultation reviews.

Unlike the ROBUST I and ROBUST III trials, this study did not require pre-dilation of the stricture before DCB use. The balloons used varied based on stricture location, with 24 Ch balloons employed for pendulous strictures and 30 Ch balloons for more proximal strictures, such as those in the bulbar, membranous, or prostatic urethra.

The primary outcome was defined as the absence of the need for reintervention following the DCB procedure, which was performed based on symptomatic burden and PVR assessed postoperatively. Secondary outcomes included the rate of postoperative complications.

The study included 43 patients, of whom 16 were treatment-naïve and 27 had recurrent urethral disease (with 11 having undergone prior urethroplasty). Stricture locations varied: 39.5% of patients had bulbar strictures. The remaining were located in the fossa navicularis (6.97%), pendular urethra (16.27%), membranous urethra (16.27%), prostatic urethra (4.65%), and bladder neck contractures (16.27%). Most strictures were iatrogenic (46.5%, without any mention of the iatrogeny mechanism), with 11.6% attributed to prior radiation therapy. Procedures were performed in both operating rooms (34.9%) and ambulatory surgical centers (65.1%).

Among the 43 patients, 90.7% (39/43) achieved the primary outcome, requiring no additional interventions during follow-up. Of the four patients who required retreatment, two were treatment-naïve, and two had recurrent disease. Two had bladder neck contractures, while the other two had a stricture on the bulbar and membranous urethra, respectively. Notably, no repeat interventions were required for patients with radiation-induced strictures (all four patients had a stricture of an iatrogenic cause).

Postoperative PVR was measured in 28 patients. Treatment-naïve patients had a mean PVR of 30.1 mL, while those with recurrent disease achieved a mean PVR of 38.1 mL, reflecting low levels of voiding dysfunction in both groups.

The safety profile was favorable, with only two immediate postoperative complications reported: one case of acute urinary retention 13 days after catheter removal requiring suprapubic tube placement and one urinary tract infection (UTI) despite negative preoperative urine culture and appropriate antibiotic perioperative prophylaxis [6]. This study was the first to examine the use of DCB outside of the original ROBUST trials, offering valuable insights into its real-world applicability.

ROBUST III: Randomized evidence supporting Optilume®'s efficacy

The ROBUST III trial is a multicenter, prospective, randomized controlled study (NCT03499964) designed to assess the safety and efficacy of the Optilume® DCB compared to standard endoscopic management, such as DVIU or dilation, for recurrent anterior urethral strictures. A total of 127 male patients with strictures ≤3 cm in length and peak urinary flow rates (Qmax) <15 mL/s were randomized in a 2:1 ratio, with 79 patients allocated to the DCB group and 48 to the standard-of-care group. Control group treatment varied: 58.3% were treated with an uncoated balloon, 25% underwent DVIU, and 16.7% underwent dilation. 

Participants were enrolled with strict inclusion criteria: anterior urethral strictures ≤12 Ch and ≤3 cm in length, at least two prior endoscopic treatments, an IPSS ≥11, and Qmax <15 mL/s. Patients with prior urethroplasty, hypospadias repair, lichen sclerosus, or unresolved etiologies (such as bladder neck contractures or benign prostatic hyperplasia) were excluded.

Pre-procedural dilation of the stricture was performed routinely in the DCB group using either an uncoated balloon (92.4%), DVIU (5.1%), or both (2.5%). The Optilume® balloon was available in various diameters and lengths (18-36 Ch; 3-5 cm) to allow customization based on patient anatomy. Surgeons were instructed to select a balloon diameter slightly larger than the adjacent healthy urethra. The DCB was inflated to the rated burst pressure for a minimum of five minutes, followed by the placement of a 12-14 Ch urethral catheter.

Post-procedural follow-ups occurred at urethral catheter removal (2-5 days), 30 days, three months, six months, and one year. Patients in the DCB cohort continued annual follow-ups for up to five years. Data collected upon each evaluation included IPSS, quality of life assessments, Qmax measurements, PVR, and freedom from reintervention analyzed through Kaplan-Meier survival curves.

At the two-year follow-up, the ROBUST III trial demonstrated significant advantages of the DCB group over standard management. Kaplan-Meier estimates showed 77.8% of DCB patients remained free from reintervention, compared to only 23.6% in the standard-of-care group (p<0.0001).

The mean Qmax increased from 7.6 mL/s at baseline to 15.5 mL/s at the one-year follow-up, nearly double the improvement compared to the control group. By two years, Qmax had slightly declined to 12.6 mL/s, suggesting that the peak improvement occurred within the first year. Nevertheless, it remained significantly above baseline.

Similarly, IPSS decreased significantly from a baseline of 22 to 9 at one year, reflecting substantial symptom relief. This initial improvement was notably greater than the control group, where IPSS began deteriorating after the three-month visit and returned to baseline levels by one year. At the two-year follow-up, IPSS was 10.1 in the DCB treatment group, slightly increased from year 1, yet still significantly lower than baseline, confirming sustained improvement.

Regarding safety, most adverse events were mild and included transitory hematuria (13.9%), dysuria (6.3%), and UTI (6.3%). Two Clavien-Dindo grade II adverse events occurred in each arm but were unrelated to the procedure (e.g., aspiration pneumonia and UTI).

Subgroup analyses revealed that even high-risk patients, that is, those with strictures ≥2 cm or a history of ≥5 prior dilations, demonstrated similar improvements in primary endpoints, with sustained benefits over two years. Importantly, erectile function was preserved throughout the study, as measured by the IIEF [7].

Ballesteros Ruiz et al.: Real-world success with Optilume®

Ballesteros Ruiz et al. conducted a retrospective, multicenter study to evaluate the efficacy and safety of the Optilume® DCB in a real-world setting. The study included 156 patients treated across 12 hospitals in Spain between May 2021 and April 2024, providing a broader perspective compared to the controlled environments of the ROBUST trials. Unlike these trials, the study's cohort represented a diverse group, with individuals with more complex clinical profiles. 

In fact, the study included patients with posterior urethral strictures (18 cases, 11.6%), with failed urethroplasty (26 cases, 16.7%), and who had undergone pelvic radiotherapy (20 cases, 12.8%). Additionally, a significant subgroup of 29 patients (18.5%) had not been submitted to any prior endoscopic intervention. The inclusion of such varied profiles offered valuable insights into the device's performance in scenarios not typically addressed in clinical trials.

In this study, treatment success was defined as a Qmax greater than 10 mL/s and the absence of need for urethral interventions (triggered by worsening voiding symptoms) during the first year of follow-up. At the six-month follow-up, the success rate was reported at 73.8%, closely aligned with the outcomes observed in the ROBUST III trial (74.6%). Functional improvements were also evident, with Qmax increasing by 8.9 mL/s at six months and 7.3 mL/s at one year. These results are consistent with those of the ROBUST III trial, which reported Qmax improvements of 7.9 mL/s at one year and 5 mL/s at two years.

Lower urinary tract symptoms, as measured by the IPSS, also showed significant improvement: IPSS scores dropped from an average baseline of 24 to 6 at six months, an 18-point improvement, which was more pronounced than the 13-point improvement reported in the ROBUST III trial at one year.

The study highlighted a favorable safety profile for Optilume®, with adverse events occurring in 14.2% of patients. The most common complications included UTI and acute urinary retention, with eight and seven cases, respectively. 

Notably, only 25% of patients who had previously undergone radiotherapy developed restenosis. The authors also state that failure rates were not related to stricture location and found no difference in the rate of restenosis in those without previous DVIU versus those who had previous manipulation.

Another important finding was that the DCB procedure was performed under local anesthesia in approximately one-third of patients. This approach demonstrated no increase in complications or failure rates, suggesting that the procedure could be feasibly performed in outpatient or clinic settings [8]. 

Table 1 provides a summary of data from the ROBUST I and III studies, as well as from studies by Mahenthiran et al. and Ballesteros Ruiz et al. 

**Table 1:** Comparison between ROBUST I, Mahenthiran et al., ROBUST III, and Ballesteros Ruiz et al. studies Ch: Charrière; DCB: drug-coated balloon; DVIU: direct visual internal urethrotomy; IPSS: International Prostate Symptom Score; PVR: post-void residual volume; Qmax: urinary peak flow rate; UTI: urinary tract infection

	ROBUST I	Mahenthiran et al.	ROBUST III	Ballesteros Ruiz et al.
Study design	Prospective, multicenter, single-arm, open-label trial	Retrospective, single-center trial	Prospective, multicenter, single-blind, randomized controlled trial	Retrospective, multicenter trial
Sample size	53 patients	43 patients	127 patients	156 patients
Inclusion criteria	Adult men with a single bulbar urethral stricture with lumen <12 Ch and length ≤2 cm, confirmed by retrograde urethrogram or cystoscopy, with a history of failed 1-4 DVIU or dilation. Patients also needed to demonstrate Qmax <10 mL/s or IPSS ≥13	Urethral strictures (<2 cm) of any location and due to any etiology were included, regardless of prior intervention	Adult men with an anterior urethral stricture ≤12 Ch and ≤3 cm in length measured by urethrogram, at least two prior endoscopic treatments, IPSS ≥11, and Qmax <15 mL/s	All patients treated with DCB in Spain between May 2021 and April 2024
Exclusion criteria	History of pelvic radiation, prior urethroplasty, radical prostatectomy, lichen sclerosus, penile prosthesis, artificial urinary sphincter, history of chronic kidney disease (creatinine >2 mg/dL), received intradetrusor onabotulinumtoxinA injections within the previous 12 months or passage of a calculus within the previous 6 months	-	Prior urethroplasty, hypospadias repair, lichen sclerosus, or unresolved confounding etiologies such as bladder neck contracture or benign prostatic hyperplasia	Follow-up <3 months
Follow-up duration	5 years	9 months	2 years	1 year
Primary endpoint	Functional success: ≥50% improvement in IPSS compared to baseline without retreatment (58%). Anatomic success: maintained urethral patency confirmed through imaging or cystoscopy (70%)	Primary outcome: absence of need for repeat intervention (90.7%) (39/43)	Kaplan-Meier estimate for freedom from reintervention: 77.8% of patients in DCB arm vs. 23.6% in the control group (p<0.0001)	Treatment success: (Qmax) >10 mL/s and absence of urethral manipulation after Optilume® due to worsening voiding quality (73.8%)
Qmax improvement	=+14.9 mL/s (5 to 19.9 mL/s) (p<0.01)	-	=+5 mL/s (7.6 to 12.6 mL/s) (p<0.003)	=+7.3 mL/s (6.3 to 13.6 mL/s)
IPSS improvement	-18 (25.2 to 7.2) (p<0.001)	-	-11.9 (22 to 10.1) (p<0.001)	-18 (24 to 6)
Reintervention rate	~28% (15/53)	~9.3% (4/43)	~22.2%	26.2%
Complications	Mild hematuria, dysuria (9%)	Hematuria, dysuria (7%)	Hematuria (13.9%), dysuria (6.3%), and UTI (6.3%)	In 14.2% of patients (UTI, eight patients; acute urinary retention, seven patients; hematuria, five patients)

Discussion

Urethral stricture is a prevalent urological condition characterized by the narrowing of the urethral lumen, leading to urinary obstruction and significant reductions in quality of life. Affecting approximately 0.6% of men during their lifetime, strictures most commonly involve the anterior urethra, with the bulbar and penile segments being the primary sites. The etiology is diverse, with iatrogenic injuries and idiopathic factors accounting for the majority of cases, although infections, trauma, and inflammatory conditions also contribute [1,9].

Despite advancements in surgical techniques, managing recurrent anterior urethral strictures remains challenging. Although urethroplasty is the gold-standard treatment, due to its high success rates, ranging from 60% to 90% depending on stricture characteristics, it is highly invasive, is associated with prolonged recovery, and requires significant postoperative care. Moreover, urethroplasty is predominantly performed in high-volume academic centers, making it less accessible to patients treated in community settings. Another critical limitation is the risk of sexual side effects, such as erectile dysfunction and penile shortening, which, while less common in contemporary studies, remains a significant concern for many patients [10-12]. These factors often drive urologists to prefer endoscopic management options like DVIU or dilation, albeit their limited efficacy and high recurrence rates [13-23]. 

In this context, the Optilume® DCB offers a novel approach to addressing these challenges. Combining mechanical urethral dilation with the localized delivery of paclitaxel, an antimitotic agent that inhibits stricture recurrence by reducing cell proliferation, Optilume® provides a minimally invasive alternative that aims to bridge the gap between endoscopic management and urethroplasty. The device was officially approved by the Food and Drug Administration (FDA) in December 2021 and included on the American Urological Association (AUA) guidelines as an adjunct to dilation or DVIU for the treatment of recurrent bulbar urethral strictures measuring less than 3 cm (conditional recommendation; evidence level: Grade B) [24].

On the other hand, in Europe, Optilume® has yet only received CE marking, meaning compliance with stringent safety, health, and environmental protection standards. Furthermore, no European or American urological society guidelines currently provide explicit recommendations for its use in treatment-naïve stricture disease, as most available evidence (including the ROBUST trials) exclusively evaluates its outcomes in patients with recurrent disease.

The efficacy of Optilume® has been demonstrated across several key studies. The ROBUST I trial, with a five-year follow-up, showed that 58% of patients achieved functional success, with significant improvements in urinary flow (Qmax), symptom scores (IPSS), and quality of life. However, the trial had strict inclusion criteria, limiting its applicability to a highly selected population [5]. In contrast, studies by Mahenthiran et al. and Ballesteros Ruiz et al. evaluated broader and more heterogeneous populations, including patients with radiation-induced strictures, treatment-naïve cases, and those who had failed previous urethroplasty [6,8]. Both studies reported comparable outcomes to ROBUST I, suggesting that Optilume® may be effective in more diverse clinical settings. Nonetheless, the small sample sizes of these subgroups limit definitive conclusions, underscoring the need for further research.

Additionally, the ROBUST III trial, the first randomized controlled study of Optilume®, reinforced its efficacy and safety. It demonstrated a significantly higher freedom from reintervention compared to standard endoscopic management (77.8% vs. 23.6% at two years) [7]. However, similar to ROBUST I, its patient population excluded individuals with complex strictures or unfavorable prognostic factors, such as lichen sclerosus or prior radiation therapy. This limitation reduces the broader application of its findings to more complex or high-risk cases.

In terms of safety, Optilume® has consistently demonstrated a favorable profile. Across all studies, complications were rare and generally mild, including dysuria and hematuria, which were transitory. No severe complications were directly linked to the procedure, reinforcing its position as a safe treatment option for recurrent urethral strictures.

Nevertheless, it is important to highlight that many of the studies reporting favorable outcomes with Optilume® incorporated pre-dilation or DVIU prior to the DCB procedure. This raises the question of whether the improved results observed in the DCB groups, especially when compared to standard endoscopic management, are partly attributable to the mechanical disruption of the stricture during pre-treatment, rather than the drug effect alone. Future studies should aim to better delineate the isolated therapeutic impact of paclitaxel-coated balloon dilatation by controlling for these procedural variables.

Moreover, another challenge in comparing studies of Optilume® lies in the variation in the definition of treatment success. While some studies emphasize anatomical success, others prioritize functional success based on symptom improvement and quality of life [6,25]. This inconsistency, aligned with different procedural techniques (e.g., pre-procedural dilation, DVIU, or sometimes both), compromises direct comparisons and underscores the need for standardized outcome measures and surgical techniques, as well as protocols in future research.

An additional insight comes from the study by Ballesteros Ruiz et al., where approximately one-third of the procedures were performed in outpatient settings under local anesthesia. Notably, this approach did not result in higher complication or failure rates, suggesting that the DCB procedure might be safely and effectively implemented outside the operating room [8]. However, Elterman et al. caution that first-time users of Optilume® may benefit from performing the procedure in an operating room with the patient under general anesthesia, as this may help minimize patient discomfort and allow surgeons to become accustomed to the technique and workflow [4].

Other advantages of Optilume® are its early post-procedural recovery and the minimal impact on sexual health. Following DCB treatment, patients may resume normal routine activities and sexual intercourse after 1-2 weeks. However, due to the presence of paclitaxel in semen for up to six months, protected intercourse is recommended for patients with partners of childbearing potential [26]. This is in contrast to urethroplasty, where sexual activity is typically restricted for six weeks or more, adding to the overall burden of recovery. Additionally, DCB treatment involves shorter catheterization times, averaging 2-5 days compared to 7-10 days for urethroplasty, further improving the patient experience and quality of life [26,27].

Economic analyses further highlight the benefits of Optilume®. Kelly et al. demonstrated significant cost savings over a five-year horizon in the NHS: £2,502 per patient compared to endoscopic management and £243 compared to urethroplasty. These savings were primarily attributed to reduced reintervention rates [28]. Similarly, the ROBUST III trial acknowledged that although pre-dilation in the DCB arm increased upfront costs, the long-term reduction in reinterventions supports its cost-saving potential, further validating Optilume® as a cost-effective alternative [7].

It is worth noting as well that the European Association of Urology Research Foundation is currently conducting a prospective, single-arm, open-label, multicenter, observational registry study (NCT05479422). This study evaluates the real-world application of Optilume® in patients with recurrent anterior urethral strictures measuring less than 3 cm. The trial includes patients treated between October 2022 and July 2024, and its findings may offer valuable insights into the device's effectiveness in broader clinical practice.

Finally, clarifying the therapeutic positioning of Optilume® remains a critical challenge. It is still uncertain whether the device should be viewed as a substitute for traditional endoscopic methods, a fallback option after failed urethroplasty, or a potential first-line alternative to open surgery in carefully selected patients. The absence of direct comparative studies between Optilume® and urethroplasty leaves a significant gap in current evidence. Determining its optimal placement within the treatment hierarchy will require head-to-head trials and long-term outcome data.

## Conclusions

Optilume® offers a minimally invasive and cost-effective solution for recurrent anterior urethral strictures, with proven efficacy and safety. Future research should focus on larger, more diverse cohorts, including complex cases like radiation-induced strictures and failures after urethroplasty. It is essential to standardize treatment success definitions as well as the procedure technique. Investigating its long-term outcomes in treatment-naïve patients and conducting tailored cost-effectiveness analyses could further support its widespread adoption and optimize its clinical use.
